# Effect of pressures and type of ventilation on aerosol delivery to chronic obstructive pulmonary disease patients

**DOI:** 10.1186/s43088-022-00234-y

**Published:** 2022-04-15

**Authors:** Marina E. Boules, Nabila Ibrahim Laz, Ahmed A. Elberry, Raghda R. S. Hussein, Mohamed E. A. Abdelrahim

**Affiliations:** 1 Clinical Pharmacy Department, Faculty of Pharmacy, Beni-Suef University, Beni-Suef, Egypt; 2 Department of Chest Diseases, Faculty of Medicine, Beni-Suef University, Beni-Suef, Egypt; 3 Clinical Pharmacology Department, Faculty of Medicine, Beni-Suef University, Beni-Suef, Egypt; 4 Clinical Pharmacy Department, Faculty of Pharmacy, Modern University for Technology and Information, Cairo, Egypt

**Keywords:** Chronic obstructive pulmonary disease, oxygen therapy, Nasal cannula, Biphasic Positive Airway Pressure, Inspiratory pressure

## Abstract

**Background:**

Continuous Positive Airway Pressure (CPAP), BiPhasic Positive Airway Pressure (BiPAP), and high flow nasal cannula (HFNC) show some evidence to have efficacy in COVID-19 patients. Delivery during noninvasive mechanical ventilation (NIV) or HFNC gives faster and more enhanced clinical effects than when aerosols are given without assisted breath. The present work aimed to compare the effect of BiPhasic Positive Airway Pressure (BiPAP) mode at two different pressures; low BiPAP (Inspiratory Positive Airway Pressure (IPAP)/Expiratory Positive Airway Pressure (EPAP) of 10/5 cm water) and high BiPAP (IPAP/EPAP of 20/5 cm water), with HFNC system on pulmonary and systemic drug delivery of salbutamol. On the first day of the experiment, all patients received 2500 μg salbutamol using Aerogen Solo vibrating mesh nebulizer. Urine samples 30 min post-dose and cumulative urinary salbutamol during the next 24 h were collected on the next day. On the third day, the ex-vivo filter was inserted before the patient to collect the delivered dose to the patient of the 2500 μg salbutamol. Salbutamol was quantified using high-performance liquid chromatography (HPLC).

**Results:**

Low-pressure BiPAP showed the highest amount delivered to the lung after 30 min followed by HFNC then high-pressure BiPAP. But the significant difference was only observed between low and high-pressure BiPAP modes (*p* = 0.012). Low-pressure BiPAP showed the highest delivered systemic delivery amount followed by HFNC then high-pressure BiPAP. Low-pressure BiPAP was significantly higher than HFNC (*p* = 0.017) and high-pressure BiPAP (*p* = 0.008). No significant difference was reported between HFNC and high-pressure BiPAP. The ex-vivo filter was the greatest in the case of low-pressure BiPAP followed by HFNC then high-pressure BiPAP. Low-pressure BiPAP was significantly higher than HFNC (*p* = 0.033) and high-pressure BiPAP (*p* = 0.008). Also, no significant difference was found between HFNC and high-pressure BiPAP.

**Conclusions:**

Our results of pulmonary, systemic, and ex-vivo drug delivery were found to be consistent. The low BiPAP delivered the highest amount followed by the HFNC then the high BiPAP with the least amount. However, no significant difference was found between HFNC and high BiPAP.

## Background

Exacerbated chronic obstructive pulmonary disease (COPD) patients, requesting ventilator support, need to administer medicated aerosols [1, 2]. Aerosols are preferred as a better route that helps in managing pulmonary diseases because it is rapid, allow the use of lower doses, deliver higher doses to lungs, and lower systemic effects [2–5]. The help of noninvasive mechanical ventilation (NIV) or high flow nasal cannula (HFNC) allows faster and more enhanced clinical effects than when aerosols are given without assisted breath [6]. This is considered of great value for critically ill patients who strongly need respiratory assistance for long periods. [7] Continuous Positive Airway Pressure (CPAP) And Biphasic Positive Airway Pressure (BiPAP)) and HFNC show emerging evidence to have efficacy in COVID-19 patients [8, 9]. Ventilatory support using a facemask and nasal mask ventilation has been greatly studied, used, and found to lower intubation in 60 to 90 percent of acute respiratory failure patients [10–12]. Also, HFNC was found to reduce intubation more than NIV and conventional oxygen therapy in patients with acute respiratory failure [13]. That helps in better oxygen delivery [14].

Nasal cannulas were used as an alternative to NIV [15], with nearly the same efficacy but fewer side effects and invasion [16, 17], due to their probable disadvantages of the facemask, e.g. skin damage, eyes irritation, decreased tolerance of interface, and interrupting expectoration, food, and speech [13, 18, 19]. Firstly, the traditional nasal cannula was used at low flow rates of oxygen up to 6 L/min. Nowadays, the HFNC system is used at high flow rates with satisfying results [7, 20–22]. HFNC lessens oxygen dilution with respiratory dead space and some positive airway pressure that is provided. In addition, the heated humidification helps in secretions' clearance and reduces the risk of bronchial hyper-response symptoms [14]. Also, humidity prevents airways dehydration which causes airway bronchospasm [23]. Aerosol delivery during noninvasive mechanical ventilation (NIV) or HFNC gives faster and more enhanced clinical effects than when aerosols are given without assisted breath. The current study aimed to compare BiPAP mode, at two different pressures, with HFNC in drug delivery in an attempt to find out which would be more beneficial and to help dose adjustment when the change from one mode to another.

## Methods

### In-vivo

**Fig. 1:**
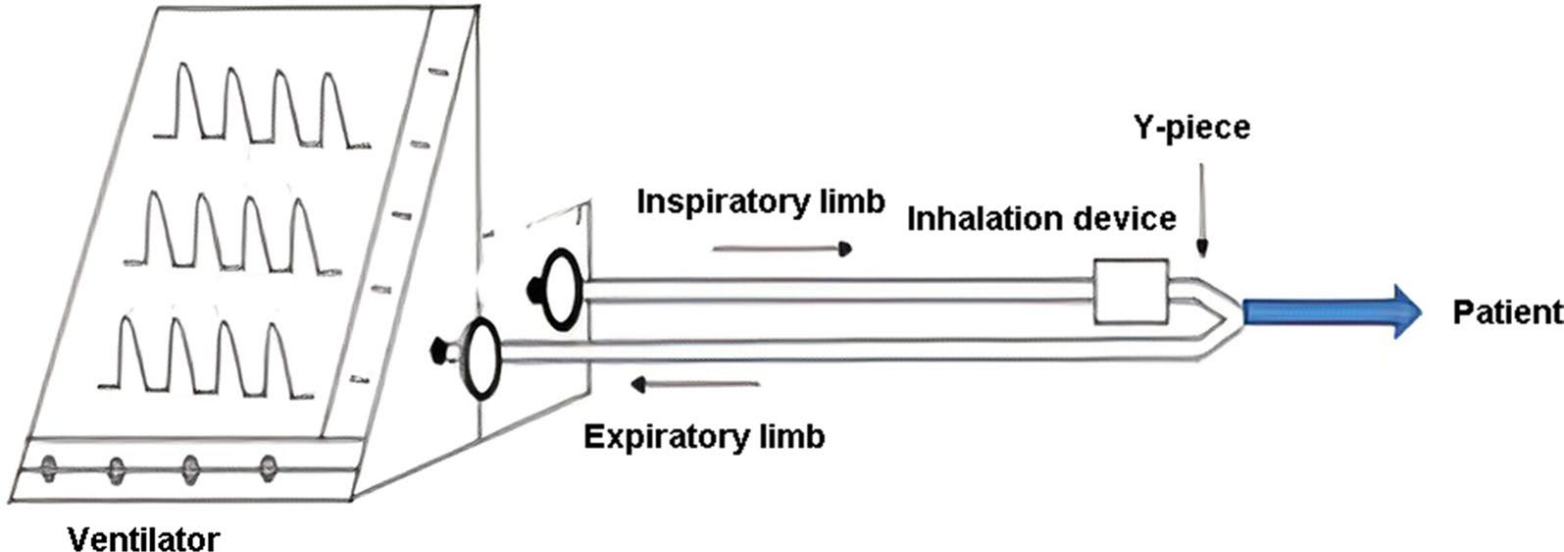
Schematic diagram of in-vivo setting showing the position of the aerosol generator in case of using BiPAP circuit. Edited from [30]

**Fig. 2:**
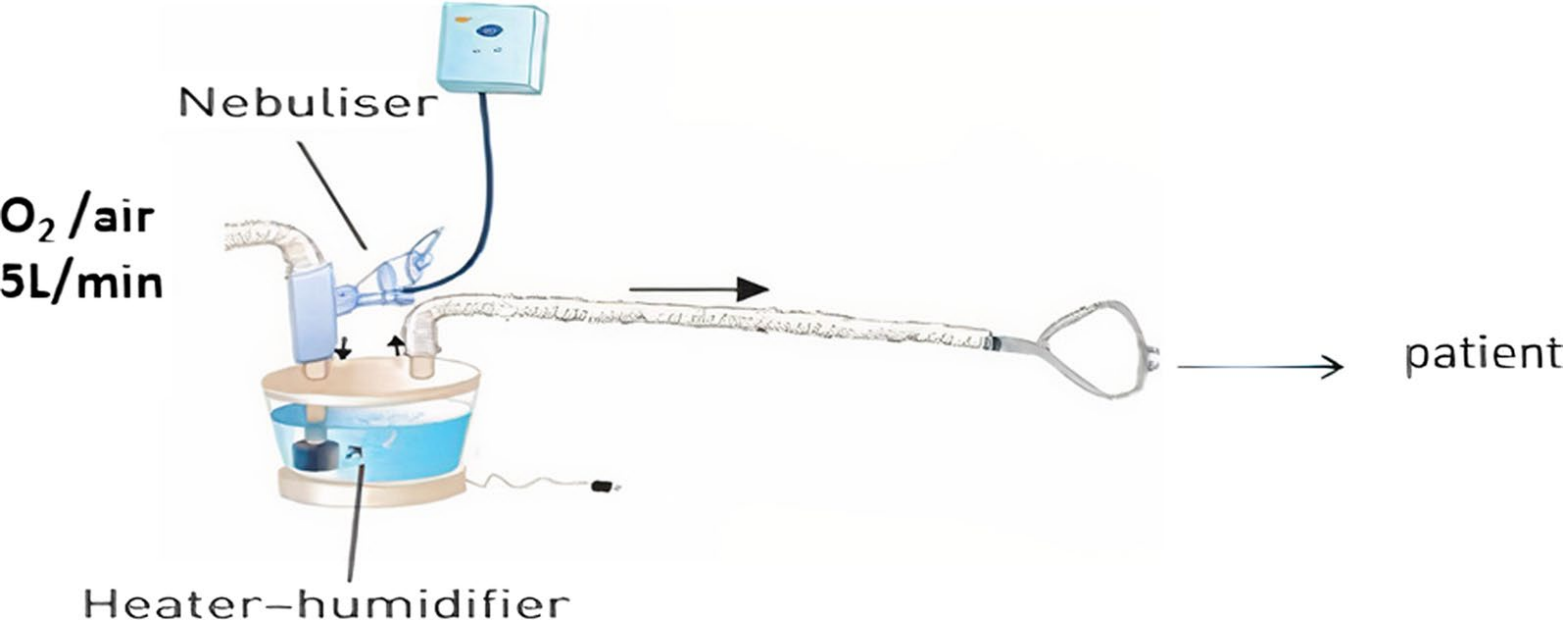
Schematic diagram of in-vivo setting showing the position of the aerosol generator in case of using HFNC circuit. [17]

The study was approved by the ''Research Ethical Committee'' of the Faculty of Pharmacy, Beni-Suef University (REC-H-PhBSU-18003). All participants signed a written informed consent. Thirty-six patients were admitted to Beni-Suef University Hospital with acute exacerbated COPD. They were randomly selected, by simple randomization using an online website (https://www.randomizer.org/). They were randomly subdivided into 3 groups; 12 patients on low-pressure BiPAP (Inspiratory Positive Airway Pressure (IPAP)/Expiratory Positive Airway Pressure (EPAP) 10/5 cm water), 12 patients on high-pressure BiPAP (IPAP/EPAP 20/5 cm water), and the 12 patients on HFNC at the flow of 5L/min. Heart Rate (HR) and Respiratory Rate (RR) were recorded before the dose. They all received 2500 μg salbutamol (Farcolin respiratory solution, 5000 μg/ml; Pharco Pharmaceuticals, Cairo, Egypt) using Aerogen Solo vibrating mesh nebulizer (SOLO; Aerogen Limited, Ireland). All the patients did not take the salbutamol dose for 48 h before the tested dose to ensure drug washout from the body. Instead, they used Ipratropium bromide (Atrovent Inhalation Solution, 2500 μg/ml, Boehringer Ingelheim, Egypt) to alleviate the bronchoconstriction. In the case of the two BiPAP groups, SOLO was inserted in the inspiratory limb (Fig. 1) [24]. The adjustment of the bi-level ventilator (Bellavista 1000e, Imtmedical, Buchs, Switzerland) was at BiPAP using the two different pressures listed above. The SOLO was inserted upstream before the humidifier (MR810 Fisher& Paykel, Fisher& Paykel Healthcare Limited, New Zealand) (Fig. 2) [25, 26] in case of using HFNC. A mixture of oxygen and room air was supplied from the gas wall supply at a low flow of 5 L/min [25, 27]. The dose was given to each patient on day 1. Urine samples were collected 30 min post-dose and cumulatively within the next 24 h post-dose indicating the pulmonary and systemic absorption, respectively. The amount of salbutamol in urine samples was collected by solid-phase extraction and then quantified by HPLC [28, 29]. The HR and RR were recorded 30 min post-dose.

On day 3, the ex-vivo study was conducted. Using the same setting except a filter placed in a filter holder (Pari GmbH, Starnberg, Germany) connected to the nasal cannula, in case of HFNC, and before the patient face mask in case of BiPAP groups. Salbutamol was collected from each filter by sonication with 20% acetonitrile and quantified by high-performance liquid chromatography (HPLC) [28, 30].

Patients were excluded if taking beta-blockers, other sympathomimetics or non-potassium sparing diuretics, pregnant females, pediatrics, having hypersensitivity to salbutamol, or suffering moderate or severe renal impairment defined as creatinine clearance or GFR of < 20 ml/min.

### Ex vivo method

The Ex-vivo study was conducted during the salbutamol wash-out period on day 2 of the study. subjects received the same dosing with the same condition performed in the in-vivo method of the previous day, but with electrostatic filter (Filta Guard breathing filter, Intersurgical, Wokingham, United Kingdom) enclosed in a filter holder (Pari GmbH, Starnberg, Germany) placed between the patient and the NIV circuit to entrap the whole inhalable dose that could reach the subject.

In the in-vivo and ex-vivo methods, nebulization was continued till no aerosol detected. Salbutamol, collected on the filter and pointed out as the total emitted dose (TED), was recovered with sonication and rinsing the filter with 25% acetonitrile (Sigma-Aldrich Chemie Gmbh, Steinheim, Germany). All collected samples were assayed by HPLC. A 4.6 × 25 mm Zobrax Eclipse as well as C18, ODS1 column (Agilent) was utilized. A mobile phase composing of a mix of acetonitrile and water with 0.1% orthophosphoric acid (90:10 v/v) was pumped through the column at a flow rate of 1 ml/min by Infinity preparative pump (G1361A, Agilent 1260). Infinity photodiode array detector VL (Agilent 1260, G131SD) was set at 225 nm with 100 µl as an injection volume. Calibration solutions from 4 to 100 µg/ml (w/v) were provided. The detection limit was 0.35 µg/ml, while the lower limit of quantification was 2.55 µg/ml [28].

### Outcome measurements

The primary outcome was the measurement of the salbutamol delivered to the lung and the body. The secondary outcome was dose adjustment when changes from one pressure to another on BiPAP or when using a nasal cannula.

### Statistical analysis

The target sample size was calculated with the power of study of 0.95, an effect size of 0.69, and an alpha value of 0.05 in which a total sample size of 36 patients (12 patients in each group) was determined as a minimum sample size using G Power.) [31].

All data are expressed as mean ± SD. One-way analysis of variance (ANOVA) with the application of least significant difference (LSD) correction was used to compare the drug delivery (30 min, 24 h urine samples, and ex-vivo delivery), baseline and 30 min post-dose patients' HR and RR and the change in HR between the three different techniques. Kruskal–Wallis Test was used to compare the change in RR between the three techniques. Paired t-test was used to compare the 30 min post-dose HR and RR to the baseline ones. All tests were done with SPSSV17.0 (SPSS Inc., Chicago, USA). Statistical significance was taken at a 95% confidence interval (*p* ≤ 0.05).

## Results

Forty-four patients participated in the study, eight of them did not complete the study, and thirty-six patients completed it. Their body mass index (BMI) and age expressed as mean ± SD are shown in Table.1. No significant difference was found in the patients' BMI or age.

**Table 1:** Body Mass Index (BMI) (kg/m^2^) and age (years) of the patients who participated in the study (*n* = 12). Values are expressed as mean ± SD

Delivery method	BMI	Age
Low BiPAP	26.49 ± 5.68	63.25 ± 6.28
High BiPAP	25.68 ± 5.46	64.75 ± 3.44
HFNC	29.07 ± 3.71	60.25 ± 8.55

Table 2 shows the baseline and 30 min post-dose HR and RR using each technique. No significant difference was found between the three modes in the baseline or 30 min post-dose HR or RR. HR significantly increased and RR significantly decreased (*p* < 0.001), in all three modes 30 min post-dose compared to baseline. Low BiPAP decreased the RR (mean ± SD 3.33 + 1.07) followed by HFNC (mean ± SD 3.25 + 0.96) then high BiPAP (mean ± SD 2.92 + 0.79) with no significant difference. Low BiPAP increased HR the most (mean ± SD 3.33 + 1.49) followed by high BiPAP (mean ± SD 3.25 + 1.76) then HFNC (mean ± SD 2.58 + 0.90) with no significant difference between the three modes.

**Table 2:** Baseline and 30 min post-dose heart rate (HR) (beat/min) and respiratory rate (RR) (breath/min) of the patients who participated in the study (*n* = 12)

Delivery method	Baseline HR (beat/min)	30 min HR (beat/min)	*p*-value	Baseline RR (breath/min)	30 min RR (breath/min)	*p*-value
Low BiPAP	83.00 ± 11.23	86.33 ± 12.40	< 0.001	23.83 ± 3.69	20.50 ± 2.94	< 0.001
High BiPAP	86.17 ± 21.20	89.42 ± 22.85	< 0.001	22. 83 ± 2.72	19.92 ± 2.43	< 0.001
HFNC	90.08 ± 4.72	92.67 ± 4.98	< 0.001	22.92 ± 1.73	19.67 ± 1.61	< 0.001

As shown in Table 3, low-pressure BiPAP showed the highest amount delivered to the lung after 30 min followed by HFNC then high-pressure BiPAP. But the significant difference was only observed between low and high-pressure BiPAP modes (*p* = 0.012). Concerning the systemic delivery, low-pressure BiPAP showed the highest delivered amount followed by HFNC then high-pressure BiPAP as illustrated in Table 3. Low-pressure BiPAP was significantly higher than HFNC (*p* = 0.017) and high-pressure BiPAP (*p* = 0.008). No significant difference was reported between HFNC and high-pressure BiPAP.

**Table 3:** The amount of salbutamol (µg) collected in urine samples 30 min and within 24 h post sample and on ex-vivo filter (*n* = 12). Values are expressed as mean ± SD

Amount of salbutamol (µg)	30 min	Within 24 h	ex-vivo filter
Low BiPAP	23.30 ± 8.31	272.07 ± 44.37	1051.29 ± 60.86
High BiPAP	16.22 ± 42.99*	162.20 ± 49.89*	747.64 ± 126.215*
HFNC	18.55 ± 5.91	173.68 ± 35.09*	812.80 ± 105.35*

Values are expressed as mean ± SD

*Significant compared with low BiPAP

Table 3 shows that the amount of drug collected on the ex-vivo filter was the greatest in the case of low-pressure BiPAP followed by HFNC then high-pressure BiPAP. Low-pressure BiPAP was significantly higher than HFNC (*p* = 0.033) and high-pressure BiPAP (*p* = 0.008). Also, no significant difference was found between HFNC and high-pressure BiPAP.

## Discussion

Drug delivery during oxygen therapy can offer great help to patients who may be affected if the circuit is disconnected and improve tolerance [25, 26, 32, 33]. Also, when the drug is delivered during assisted breathing, it shows enhancement in the clinical effects in a faster way [5, 7]. The SOLO nebulizer was inserted in the Y limb, in case of using BiPAP modes, to provide the highest delivery [24, 29].

In the current study, the results of pulmonary, systemic, and ex-vivo drug delivery were all found to be consistent. Generally, low BiPAP delivered the highest amount followed by HFNC then high BiPAP with the least amount. However, no significant difference was found between HFNC and high BiPAP in drug delivery. These results of the present study match the results of our in-vitro study (in press) in which the total inhalable dose(TID) and Fine Particle Dose (FPD) were the greatest using low BiPAP followed by HFNC then high BiPAP with the least amount [34].

Increasing IPAP caused a reduction in the amount of drug delivered either to the lung, systemic circulation or deposited on an ex-vivo filter. This is supported by the results found by Velasco and Berlinski [24] who found that increasing IPAP decreased the drug delivery efficiency either if SOLO was inserted before the mask, before the Y-piece, and at the ventilator.

In contrast, Chatmongkolchart, et al. [35] revealed that increasing IPAP increased the drug delivery if a nebulizer was inserted distal from the BiPAP ventilator (proximal to the lung model). This may be due to the usage of a single limb ventilator with the nebulizer inserted between the exhalation port and the lung model in their study. Consequently, there was a retrograde return. However, in the same study of Chatmongkolchart, et al., in accordance with our study, they reported that increasing BiPAP decreased the drug delivery [36].

L'Her et al. found that oxygenation improved when positive end-expiratory pressure (PEEP) was increased from 5 to 10 cm H_2_O, also dyspnea showed the best enhancement by increasing pressure support (PS) from 10 to 15 cm H_2_O [37]. However, their study did not include quantification of the drug delivered.

Pressure support ventilation (PSV) is a positive airway pressure, detected by a clinician, assisted by a mechanical ventilator for the patient's spontaneous inspiratory efforts like IPAP [38]. L'Her et al. found that when PS increases, dyspnea is improved as mentioned formerly. In the present study, using IPAP generally improved RR, but there was no significant difference between low and high BiPAP in improving RR.

The PEEP is the positive pressure that remains in the airways at the end of exhalation [39] like EPAP. It helps in recruitment and stabilization of collapsed lung tissue [37, 40], a decrease of alveolar stress [40] and the effort required of mechanically ventilated patients [41] and enhancement of gas exchange [42] so enhances oxygenation [43, 44]. This can be due to that sufficient PEEP helps to evacuate the circuit from the expired CO_2_, preventing rebreathing, out to the atmosphere with the aid of enough time of expiration [45].

Consequently, there must be a careful choice whether to increase the PEEP level for enhanced oxygenation or to increase the PSV level for improved dyspnea and reduced respiratory muscle effort [37]. So, we recommend further studies comparing the effect of increasing EPAP while holding IPAP.

The HFNC system was earlier found to enhance all respiratory parameters and oxygenation and be well tolerated when used for long periods than traditional facemasks [14, 46]. The more the oxygen flow in the HFNC system, the better the oxygenation would be [14, 47].

Both Ari et al. [27] and Perry et al. [32] found that increasing the flow of the HFNC system caused a reduction in the drug delivery and the best flow that was found for adults was 5 L min^−1^. So, in the current study, oxygen was delivered at that low flow of 5 L min^−1^.

The HFNC system, compared to low BiPAP, delivered a lower amount of the drug. The heated humidified circuit used in HFNC leads to aerosol condensation within the circuit and loss which was augmented by the smaller diameter and longer length of nasal cannula over the BiPAP circuit [26, 32, 48, 49]. Also, when comparing nasal to mask delivery, aerosol particles are filtered more efficiently through the nose, than the mouth, leading to a reduction in the dose available to penetrate the lower respiratory tract [50]. In addition, the turbulent gas flow in the nose and rhino-pharynx may favor drug deposition decreasing the amount of drug that can reach the lungs [25, 49].

On the other hand, deposition of the large particles of aerosol in the HFNC circuit decreases the delivered dose, improves tolerance. In this way, it decreases the deposition of these large particles on the face (potentially including eyes) and upper airways which happens when using facemasks [51]. Also, it shows a better-tolerated technique than facemasks [52] which may cause the feeling of confining, coldness, irritation, preventing communication and oral intake, that may be needed to act as a worn for long periods that may lead to, especially in children, fussing, crying, and screaming so reduced aerosol lung deposition, unlike HFNC circuit, humidified and heated conditions which improves patient comfort, may improve lung deposition and increase tolerance to use for long periods [51, 52].

In the present study, the percentage of the amount of drug delivered to the patient by low BiPAP, high BiPAP, and HFNC after 30 min were 0.932, 0.649, and 0.742% of the nominal dose, respectively. The cumulative percent of the amount within 24 h delivered to the patient by low BiPAP, high BiPAP, and HFNC were 10.883, 6.488, and 6.947% of the nominal dose, respectively. The former data were found to be greatly lower than the percentage of the amount collected from the ex-vivo filter; 42.052, 29.906, and 32.512% of nominal dose delivered by low BiPAP, high BiPAP, and HFNC, respectively. That could be because the particles less than 1 µm cannot deposit in the lung unless the patients hold his breath for 5–10 s [32, 53, 54] and since this patient could not make a 5–10 s breath-hold to get deposited most of the aerosol less than 1 µm was exhaled [7]. Also, the aerosol particles produced by the SOLO of 5 μm and above decreases the percent that would reach the lung as particle sizes of 1–3 μm [54–56].

Although low BiPAP delivered the highest amount of aerosol to the lung indicating better efficacy, it also delivered the highest amount systemically, so more side effects can occur to the patients [1]. The results of the current study can help in dose adjustment when changing from one technique to another. Depending on pulmonary drug delivery results, the amount of salbutamol delivered to the lung using 2.50 mg salbutamol on low BiPAP mode was equivalent to the amount delivered using 3.59 mg salbutamol on high BiPAP mode and to the amount delivered using 3.14 mg salbutamol on HFNC mode. Depending on systemic drug delivery results, the amount of salbutamol delivered to the body using 2.50 mg salbutamol delivered on low BiPAP mode was equivalent to the amount delivered using 4.19 mg salbutamol on high BiPAP mode and to the amount delivered using 3.92 mg salbutamol on HFNC mode.

Consequently, dose adjustment guidelines must be developed to be used when changing from one technique to another. If similar doses are used, there may not be of clinical difference in the bronchodilation but patient safety may be affected due to change in systemic delivery [29].

In accordance with the present study results, the HFNC reduced respiratory rate efficiently in Corley et al. [57], Bell et al. [58], Makdee et al. [59] studies. Also, Sztrymf et al. [46] reported that breathing frequency decreased significantly when using HFNC. In addition, Vargas et al. [60] revealed breathing frequency decrease too when HFNC was used in acute hypoxemic respiratory failure, which can be attributed to the reduction of breathing work and enhancement of oxygenation.

The present study results showed that all the three techniques improved RR significantly from the baseline, but no significant difference was found between the three techniques. On the other hand, in Schwabbauer et al. studied [61], HFNC significantly improved RR than NIV. This can be attributed to the difference in the conditions from the current study. They used HFNC at gas flow 55 L/min which was much greater than the current study.

## Limitations

Spirometry pre- and post-dose could not be measured because interruption of ventilator support during mechanical ventilation is not possible in our institution.

## Conclusions

Aerosols delivery simultaneously during NIV or HFNC can help greatly in patients who may be affected if the circuit is disconnected and improves tolerance. HFNC was found to improve tolerance and give comparable results to low-pressure BIPAP mode. Increasing IPAP was found to decrease both the pulmonary and systemic delivered dose. Consequently, there must be a careful dose adjustment when changing the pressure levels used in BiPAP or when using the HFNC system. All three techniques were found to improve RR significantly from baseline dose, but no significant difference was found between the three techniques. Further studies are recommended to test the effect of increasing EPAP on the amount of delivered dose.

## Data Availability

The datasets analyzed during the current study are available from the corresponding author on reasonable request.

## Abbreviations

BiPAP: BiPhasic Positive Airway pressure
IPAP: Inspiratory Positive Airway Pressure
EPAP: Expiratory Positive Airway Pressure
NIV: Noninvasive mechanical ventilation
HFNC: High flow nasal cannula
HPLC: High-performance liquid chromatography
COPD: Chronic obstructive pulmonary disease
HR: Heart Rate
RR: Respiratory Rate
SOLO: Aerogen Solo vibrating mesh nebulizer
BMI: Body mass index
